# Orange peel-mediated synthesis of silver nanoparticles with antioxidant and antitumor activities

**DOI:** 10.1186/s12896-024-00892-z

**Published:** 2024-09-27

**Authors:** Bardees Mickky, Heba Elsaka, Muhammad Abbas, Ahmed Gebreil, Reham Shams Eldeen

**Affiliations:** 1 Botany Department, Faculty of Science, Mansoura University, Mansoura, 35516 Egypt; 2 Botany & Microbiology Department, Faculty of Science, Arish University, Arish, 45511 Egypt

**Keywords:** Antioxidant, Antitumor, Characterization, Orange peel, Silver nanoparticles

## Abstract

Orange (*Citrus sinensis* L.) is a common fruit crop widely distributed worldwide with the peel of its fruits representing about 50% of fruit mass. In the current study, orange peel was employed to mediate the synthesis of silver nanoparticles (AgNPs) in a low-cost green approach. Aqueous extracts of suitably-processed peel were prepared using different extraction methods; and their phytochemical profile was identified. Based on phytochemical screening, amount of main phytochemicals, free radical-scavenging ability, reducing power and antioxidant activity, the peel extract prepared by boiling seemed to be the most promising. Thus, major compounds of this extract were identified by gas chromatography-mass spectrometry. Potency of the peel extract to mediate the synthesis of AgNPs was then monitored by visual observation, UV-visible spectroscopy, energy dispersive X-ray analysis, transmission electron microscopy and zetametry. Color change of the reaction mixture to brown and absorption peak at 450 nm indicated AgNPs formation. Characterization of AgNPs revealed spherical shape, size of 30–40 nm, zeta potential of -18.2 mV and yield conversion of 82%. The as-synthesized AgNPs had antioxidant capacity (free radical-scavenging ability, reducing power and antioxidant activity) lower than that of the orange peel extract. However, these biogenic AgNPs had antitumor activity (IC_50_ of 16 ppm against HCT-116 and 1.6 ppm against HepG2 cell lines) much higher than the peel extract that was completely non-toxic to the considered cell lines.

## Introduction

Navel orange (*Citrus sinensis* L.; Family Rutaceae) is one of the most common fruit crops whose medicinal and aromatic properties are known since the ancient times [1]. Fruit of navel orange is seedless and easy to peel. Its flesh is juicy with aromatic and moderately-acidic taste. Processing industry of orange, especially juice extraction, annually produces large quantities of wastes the majority of which is the peel [2]. This causes waste of resources and triggers environmental pollution while discarding such abundant resource. Therefore, considerable attention is recently paid to strategies that allow value-added utilization of orange peel [3]. These strategies depend on the extraction of bioactive compounds from the peel in easy, fast and economic way. So, the choice of extraction method and solvent are of great importance when targeting applicable approach.

Numerous studies on orange peel confirmed its astonishing phytochemical profile. In this regard, phytochemical composition of orange peel seemed to be the core reason behind its health benefits [4]. These benefits were found to rely on its antioxidant, antimicrobial and anti-inflammatory properties [5]. Nevertheless, antioxidant capacity of the bioactive compounds of orange peel is the most outstanding [6]. Phytochemicals identified in orange peel can be categorized into primary and secondary metabolites. Primary metabolites abundant in orange peel are proteins and carbohydrates [7]. Such proteins were identified as free amino acids and/or definite protein polymers [8]. Meanwhile, carbohydrates of orange peel are mainly sugars whether reducing or non-reducing [9]. However, the key secondary metabolites identified in orange peel include phenols, carotenoids and essential oils [4]. Apart from essential oils that require careful extraction procedures, flavonoids represent the greatest portion of phenolic compounds in orange peel [10]. In this regard, anthocyanins were recorded as a sub-group of flavonoids with marked reducing power and antioxidant activity [11]. Moreover, survey of literature indicated that orange peel is rich in carotenoids in the form of carotenes (alpha and beta carotenes as well as lycopene) and xanthophylls [12]. In addition, ascorbic acid is a main phyto-constituent in orange peel with well documented free radical-scavenging ability, reducing power and antioxidant activity [13].

The rich phytochemical profile of orange peel allowed it to be used for the synthesis of various nanoparticles [14, 15]. Among nanoparticles, silver nanoparticles (AgNPs) are inorganic nanomaterials that gained pronounced attention in the past few years [16]. Generally, AgNPs could be synthesized by physical, chemical and biological methods. Of these, biological methods mediated by microorganisms or plant extracts are more effective. Biogenic synthesis of AgNPs using different plant extracts proved to be simple, fast, eco-friendly and cost effective [17]. Because of their unique properties, biogenic AgNPs are involved in many applications especially in the medical field. In this regard, AgNPs were recorded to possess antimicrobial, anti-platelet and wound-healing activities [18]. Furthermore, both antioxidant and antitumor potencies of AgNPs were documented [19].

For AgNPs whose synthesis is mediated by plant extracts, it is crucial to compare antioxidant and antitumor activities of these AgNPs with those of the extracts involved in their synthesis. Hence, the current study aimed at using orange peel extracts simply prepared by different methods to synthesize AgNPs. Firstly, phytochemical profile of the peel extracts was analyzed followed by determining their antioxidant capacity. Then, AgNPs obtained with the aid of the most promising extract were characterized and their antioxidant capacity was similarly assessed. Moreover, antitumor activity of the obtained AgNPs was evaluated and compared with that of the peel extract.

## Materials and methods

### Plant material and extraction methods

**Fig. 1:**
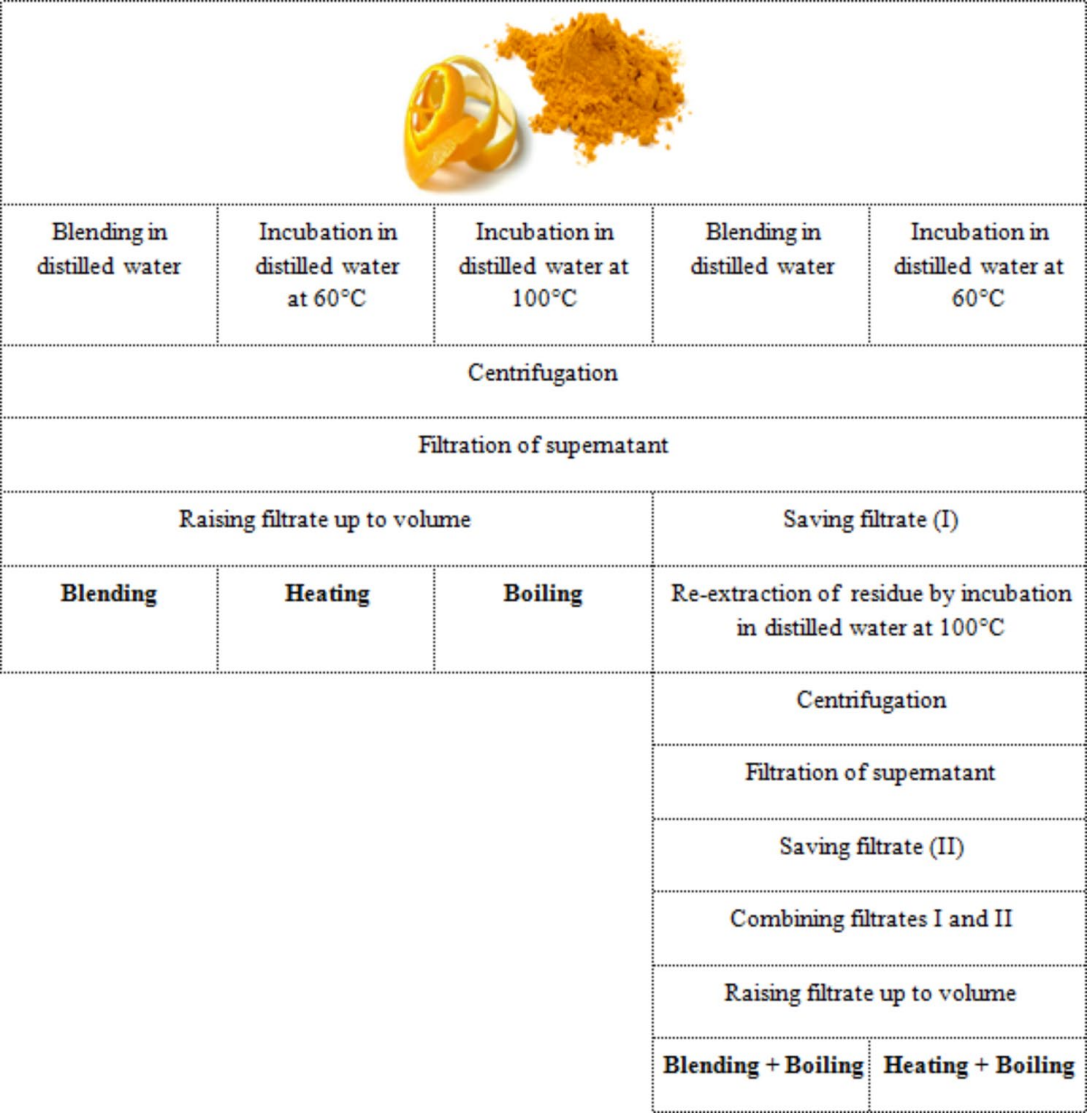
Flowchart for the five methods used to extract orange peel

Ripe fruits of navel orange (*Citrus sinensis* L. Osbeck) were purchased from local markets based on fruit botanical description introduced by Dongre et al. [20]. Fruits were washed then peeled and the peel was air dried, pulverized and sieved to fine powder. Five methods were followed to prepare 5% aqueous extracts. The first method involved blending the peel powder in distilled water for 30 min. The second and the third methods involved incubating the powder with water for 30 min in water bath at 60 °C and 100 °C; respectively. For the three methods, the homogenate was centrifuged at 4,000 rpm for 30 min then the supernatant was filtered through Whatman No. 40 filter paper and the filtrate was raised up to volume. In the fourth and fifth methods, the residue left after blending or heating was re-extracted by incubating at 100 °C for 30 min, similarly purified then the supernatants was combined with the first ones. Thus, the methods were marked as; (i) blending, (ii) heating, (iii) boiling, (iv) blending + boiling and (v) heating + boiling (Fig. 1).

### Qualitative screening of phytochemicals in orange peel extracts

The five extracts were screened for phytochemicals according to Harborne [21] and Kokate [22]. Carbohydrates were tested by α-naphthol and H_2_SO_4_, reducing sugars by Benedict reagent, proteins by NaOH and CuSO_4_, amino acids by ninhydrin, phenols by lead acetate, flavonoids by acidified NaOH, alkaloids by Mayer reagent, tannins by FeCl_3_, terpenoids by chloroform and H_2_SO_4_, steroids by acetic anhydride and H_2_SO_4_, saponins by shaking, chalcones by ammonia, anthraquinones by benzene and ammonia, coumarine glycosides by NaOH and sodium nitroprusside, while cardiac glycosides were tested by glacial acetic acid, FeCl_3_ and H_2_SO_4_. The results were expressed as high, average, moderate, low or no detected amount of phytochemicals; and represented as icon map.

### Quantitative determination of main phytochemicals in orange peel extracts

According to Ogunlesi et al. [23], the amount of ascorbic acid in the extracts was determined by titration against 2,6-dichlorophenol indophenol. According to the colorimetric methods of Sadasivam and Manickam [24], total amount of proteins was determined by coomassie brilliant blue G250, amino acids by ninhydrin, carbohydrates by phenol and sulphuric acid, reducing sugars by dinitrosalicylic acid and phenols by Folin-Ciocalteau reagent. The amount of non-reducing sugars was determined as the difference between carbohydrates and reducing sugars. Total amount of flavonoids was determined following Dewanto et al. [25] using NaNO_2_, AlCl_3_ and NaOH. Total amount of anthocyanins was determined by the formula proposed by Lees and Francis [26], carotenoids by the formula proposed by Kissimon [27], while carotenes and xanthophylls were determined by the formulae of Bulda et al. [28]. The amount of each of these phytochemicals was expressed in % (g 100 g^− 1^ peel dry weight).

### Determination of antioxidant capacity of orange peel extracts

Diphenyl picrylhydrazyl (DPPH)-scavenging ability of the five extracts was determined following Siger et al. [29]; and IC_50_ was derived graphically and expressed in g 100 ml^− 1^ peel extract. Reducing power was determined following Dorman and Hiltunen [30], while antioxidant activity was determined following Prieto et al. [31]; and the two were expressed in mg ascorbic acid equivalent (AAE) ml^− 1^ peel extract.

### Characterization of the most promising extract

Based on phytochemical screening as well as the amount of main phytochemicals and antioxidant capacity of the five extracts, the most promising one was subjected to gas chromatography-mass spectrometry (GC-MS). Agilent 6890 gas chromatograph equipped with mass spectrometric detector was used with PAS-5MS column (30 m x 0.32 mm x 0.25 μm film thickness). Helium was used as carrier gas at approximately 1 ml per minute in a pulsed split-less mode. The solvent delay was 3 min, the injection volume was 1 µl and the injector temperature was 280 °C. The mass spectrometric detector was operated in electron impact ionization mode with an ionizing energy of 70 eV scanning 50–500 m/z. The ion source temperature was 230 °C. The electron multiplier voltage was maintained at 1650 V above auto tune. The instrument was manually tuned using perfluorotributyl amine. The gas chromatograph temperature program was started at 60 °C for 2 min then elevated to 300 °C at rate of 5 °C per minute. National Institute of Standards and Technology (NIST) library was used to analyze the separated peaks.

### Biogenic synthesis and characterization of AgNPs

In firmly closed flaks, 20 ml orange peel aqueous extract prepared by boiling was mixed with 80 ml AgNO_3_ (1 mM) for 24 h in dark with continuous shaking at 120 rpm. Formation of AgNPs was monitored by visual observation as well as spectral analysis in the range of 300–800 nm using 6705 UV-visible spectrophotometer (Jenway, England). Elemental analysis of the extract and the resulted AgNPs was performed by energy-dispersive X-ray (EDX) analysis using X-ray unit (Oxford, UK) attached to scanning electron microscope (JEOL, Japan). Also, Fourier transform infrared (FTIR) spectroscopy of the extract and the resulted AgNPs was performed by Nicolet iS-10 FTIR spectrometer (Thermo Scientific, USA). Moreover, characterization of AgNPs by transmission electron microscopy (TEM) and high resolution TEM (HR-TEM) was achieved using JEM-2100 transmission electron microscope (JEOL, Japan). In addition, zetametry was carried out to determine size distribution by volume and zeta potential using Nano-zs90 zeta analyzer (Malvern, UK).

### Determination of yield, antioxidant potency and antitumor activity of AgNPs

Concentration of AgNPs was determined in ppm using atomic absorption spectrometry; and conversion % was derived based on the initial AgNO_3_ concentration. Antioxidant capacity of AgNPs was assessed by determining their IC_50_ for DPPH-scavenging ability as well as reducing power and antioxidant activity as previously described for the peel extracts. For antitumor activity, two cell lines (colorectal HCT-116 and hepatocellular HepG2) were obtained from American Type Culture Collection. Cytotoxicity of AgNPs and the peel extract prepared by boiling was assessed by 3-(4,5-dimethylthiazol-2-yl)-2,5-diphenyltetrazolium bromide (MTT) assay [32]. Cell viability was calculated in % based on cell images acquired by GXMGXD202 inverted microscope; with IC_50_ derived graphically and expressed in ppm.

### Statistical analysis

Data obtained for some determinations were statistically analyzed for standard deviation and analysis of variance (ANOVA) at *p* ≤ 0.05 using CoHort/CoStat^®^ software version 6.311.

## Results and discussion

### Qualitative screening of phytochemicals in orange peel extracts

In the current study, orange peel was extracted in distilled water as the simplest, most available and most inert solvent. Survey of literature indicated that various plant tissues were extracted by different methods. Blending, heating and boiling are common single-stepped extraction methods. However, the current study presented new trend in extraction via two double-steeped methods. Phytochemicals that may be sensitive to high temperature were firstly extracted by blending or heating at 60 °C then the residue was fully re-extracted by boiling. The obtained extracts were then subjected to in-depth phytochemical analysis.

**Fig. 2:**
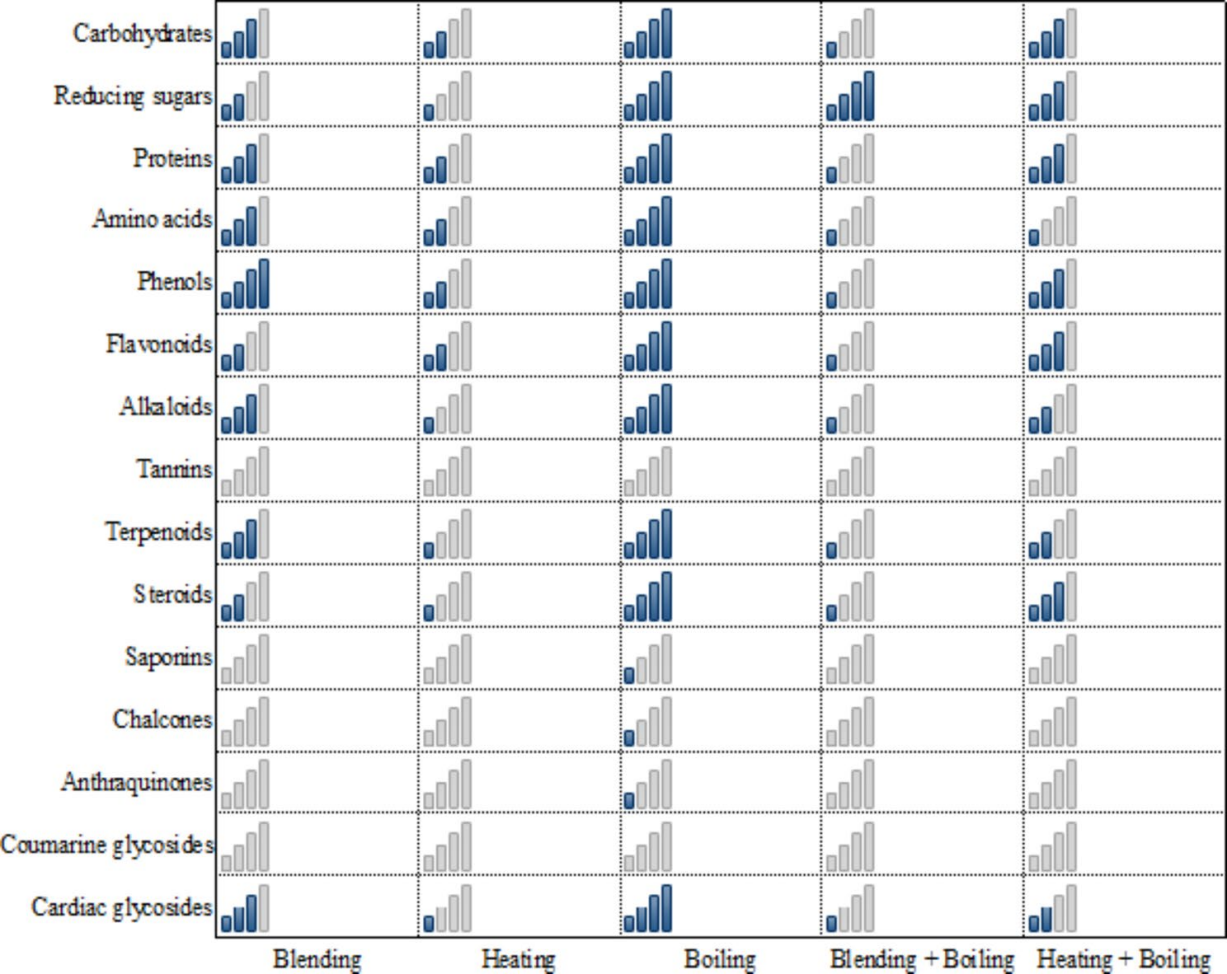
Qualitative screening of phytochemicals in orange peel aqueous extracts prepared by different methods. Number of blue bars in each cell indicates high, average, moderate, low or no detected amount of phytochemicals

Qualitative screening of phytochemicals in orange peel extracts indicated the presence of all the considered phytochemicals in the five extracts; except for tannins and coumarine glycosides that were not detected in any extract (Fig. 2). Saponins, chalcones and anthraquinones were detected only in the extract prepared by boiling. The other phytochemicals (carbohydrates, reducing sugars, proteins, amino acids, phenols, flavonoids, alkaloids, terpenoids, steroids and cardiac glycosides) were identified in all extracts; with the highest titer of these phytochemicals recorded for the extract prepared by boiling (Fig. 2). However, the extract prepared by blending was almost as the same as that prepared by boiling in showing the highest titer of phenols. Therefore, orange peel extract prepared by boiling seemed to be the most promising based on qualitative screening of phytochemicals.

In recent studies, similar phytochemical classes could be identified in orange peel [33]. Extraction of phytochemicals by organic solvents may be more efficient; but these solvents are usually expensive and environmentally harmful [34]. Thus, the use of distilled water as an extraction solvent for various plant tissues is common [35]. Matching the extraction protocols followed in the current study, Banu and Cathrine [36] pointed out to natural drying of plant tissues followed by powdering and extraction in water by various methods like maceration (parallel herein to blending) and decoction (parallel to boiling). Aqueous extraction of plant tissues by incubating at boiling water bath was previously reported as an efficient way to extract phytochemicals [37].

### Amount of main phytochemicals and antioxidant capacity of orange peel extracts

**Fig. 3:**
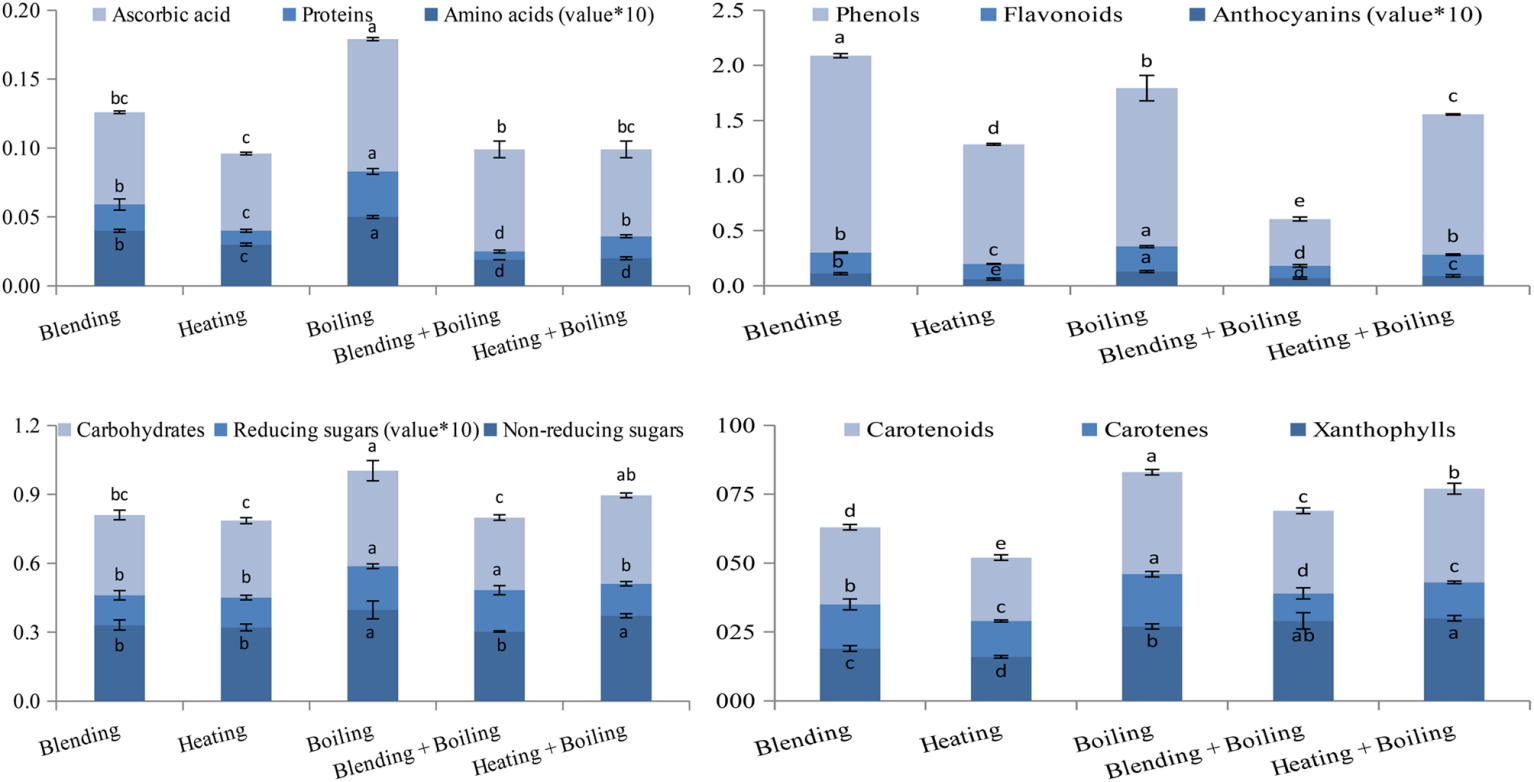
Amount of phytochemicals (%) in orange peel aqueous extracts prepared by different methods. Error bars represent standard deviation of three replicates; with different letters referring to significant difference for each parameter at *p* ≤ 0.05

**Fig. 4:**
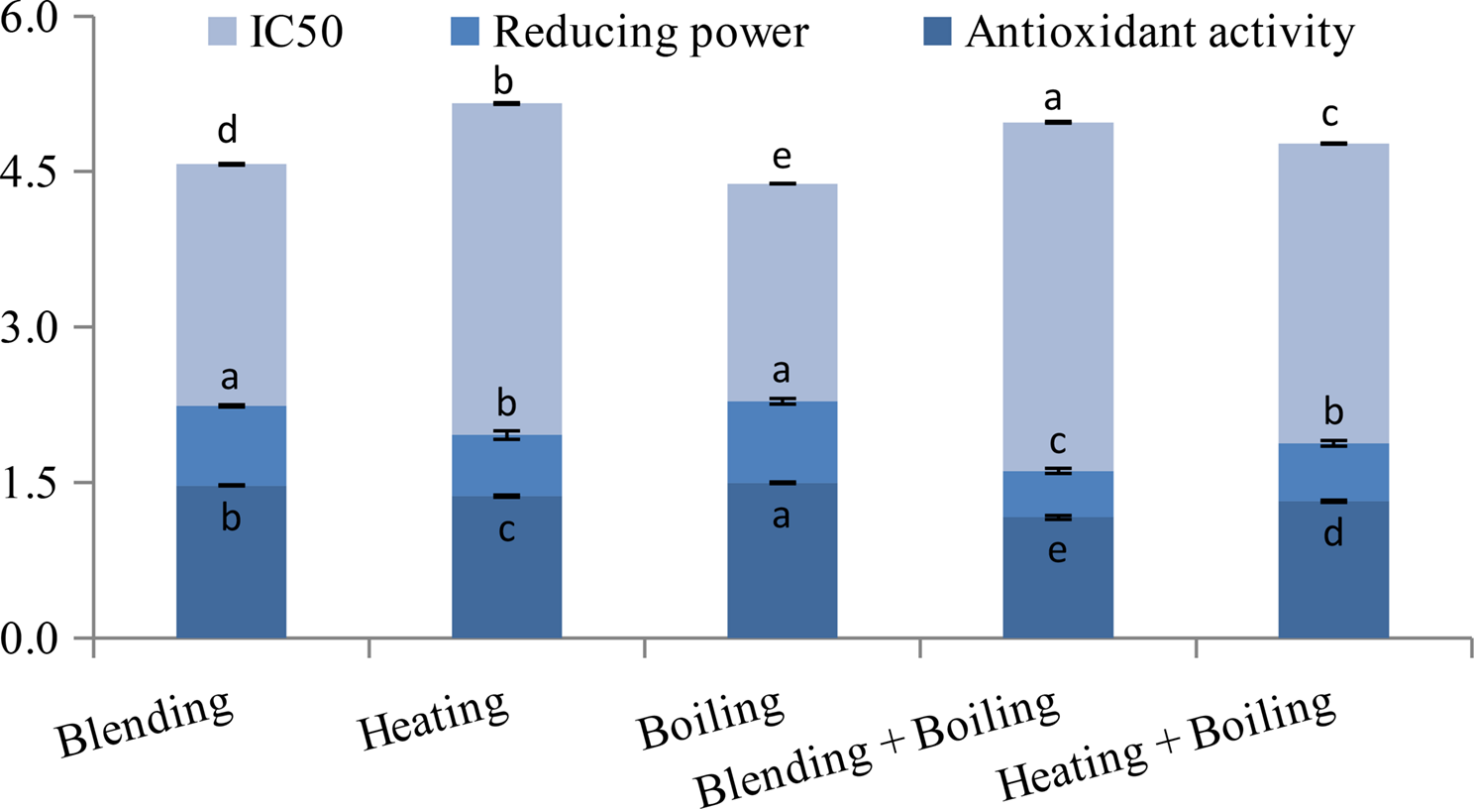
IC_50_ for DPPH-scavenging ability (g 100 ml^− 1^) as well as reducing power and antioxidant activity (mg AAE ml^− 1^) of orange peel aqueous extracts prepared by different methods. Error bars represent standard deviation of three replicates; with different letters referring to significant difference for each parameter at *p* ≤ 0.05

Among the five extracts prepared from orange peel, the extract prepared by boiling showed the maximum amount of ascorbic acid, proteins, amino acids, reducing sugars, flavonoids, anthocyanins, carotenoids and carotenes (Fig. 3). The amount of each of these phytochemicals in the extract prepared by boiling was significantly higher than that in the extracts prepared by the other methods (*p* ≤ 0.05). The maximum amount of total carbohydrates and that of non-reducing sugars were detected in the extract prepared by boiling as well as that prepared by heating + boiling (*p* ≤ 0.05). However, the maximum amount of total phenols was detected in the extract prepared by heating followed by the extract prepared by boiling (*p* ≤ 0.05). For xanthophylls, the maximum amount was detected in the extract prepared by heating + boiling (Fig. 3). Furthermore, the extract prepared by boiling possessed the maximum DPPH-scavenging ability as indicated from the lowest IC_50_ at *p* ≤ 0.05 (Fig. 4). In addition, the maximum reducing power and the maximum antioxidant activity were determined for the extract prepared by boiling. Comparable with that extract, the extract prepared by blending showed maximum reducing power at *p* ≤ 0.05 (Fig. 4). Thus, orange peel aqueous extract prepared by boiling seemed to be the most promising based on the amount of main phytochemicals and its antioxidant capacity.

Orange peel is well documented to be a natural source of ascorbic acid which is a water-soluble vitamin [13]. Ascorbic acid has potent free radical-scavenging ability, reducing power and antioxidant activity [38]. Also, orange peel is documented to contain considerable amounts of proteins and carbohydrates [39]. In a study that compared total phenol, flavonoids and anthocyanins content of five fruit peels, orange peel possessed the maximum amount of these phytochemicals compared with the other studied fruits [40]. In this regard, free radical-scavenging ability, reducing power and antioxidant activity of different plant tissues were mainly ascribed to their phenol, flavonoids and anthocyanins content [4]. Moreover, survey of literature indicated that orange peel is rich in carotenoids in the form of carotenes and xanthophylls as the main sub-classes [12]. Carotenoids are another class of phytochemicals with potent free radical-scavenging ability and antioxidant activity [41].

### Characterization of the most promising extract

Gas chromatography-mass spectrometry (GC-MS) of orange peel aqueous extract prepared by boiling indicated the presence of two fatty acids; palmitic acid and oleic acid (Table 1). These two fatty acids are documented with potent antioxidant activity [42]. Also, GC-MS indicated the presence of three phenolic compounds (4-vinylphenol, 2-methoxy-4-vinylphenol and 2,6-di-methoxy-4-vinyl-phenol) (Table 1). These compounds are documented with potent reducing power and antioxidant activity [43]. In addition, GC-MS revealed the presence of some silicone oils such as hexamethylcyclotetrasiloxane (D3), octamethylcyclotetrasiloxane (D4) and decamethylcyclotetrasiloxane (D5). Nonetheless, siloxanes are synthetic compounds; so these three compounds may be artifacts. More importantly, orange peel aqueous extract prepared by boiling was characterized by the presence of a major compound classified as organic acid or as vitamin and documented with potent free radical-scavenging ability, reducing power and antioxidant activity. This compound was identified as 1-(+)-ascorbic acid (Table 1). Confirming this result, considerable amount of ascorbic acid was determined in the peel extract prepared by boiling (Fig. 3).

**Table 1:** Major compounds revealed by GC-MS of orange peel aqueous extract prepared by boiling and used to mediate AgNPs synthesis

Retention time (min)	Peak area (%)	Compound name	CAS #	Chemical formula
30	5.10	Palmitic acid	000057-10-3	C_16_H_32_O_2_
34.192	11.45	Oleic Acid	000112-80-1	C_18_H_34_O_2_
15.088	0.53	4-vinylphenol	339241-81-5	C_12_H_16_O
16.108	31.60	2-methoxy-4-vinylphenol	007786-61-0	C_9_H_10_O_2_
22.522	7.34	2,6-di-methoxy-4-vinyl-phenol	998130-83-7	C_10_H_12_O_3_
6.669	10.89	Octamethylcyclotetrasiloxane	000556-67-2	C_8_H_24_O_4_Si_4_
10.092	9.75	Hexamethylcyclotrisiloxane	000541-05-9	C_6_H_18_O_3_Si_3_
10.677	4.27	Decamethylcyclopentasiloxane	000541-02-6	C_10_H_30_O_5_Si_5_
30.891	5.1	1-(+)-Ascorbic acid	028474-90-0	C_6_H_8_O_6_

### Biogenic synthesis and characterization of AgNPs

**Fig. 5:**
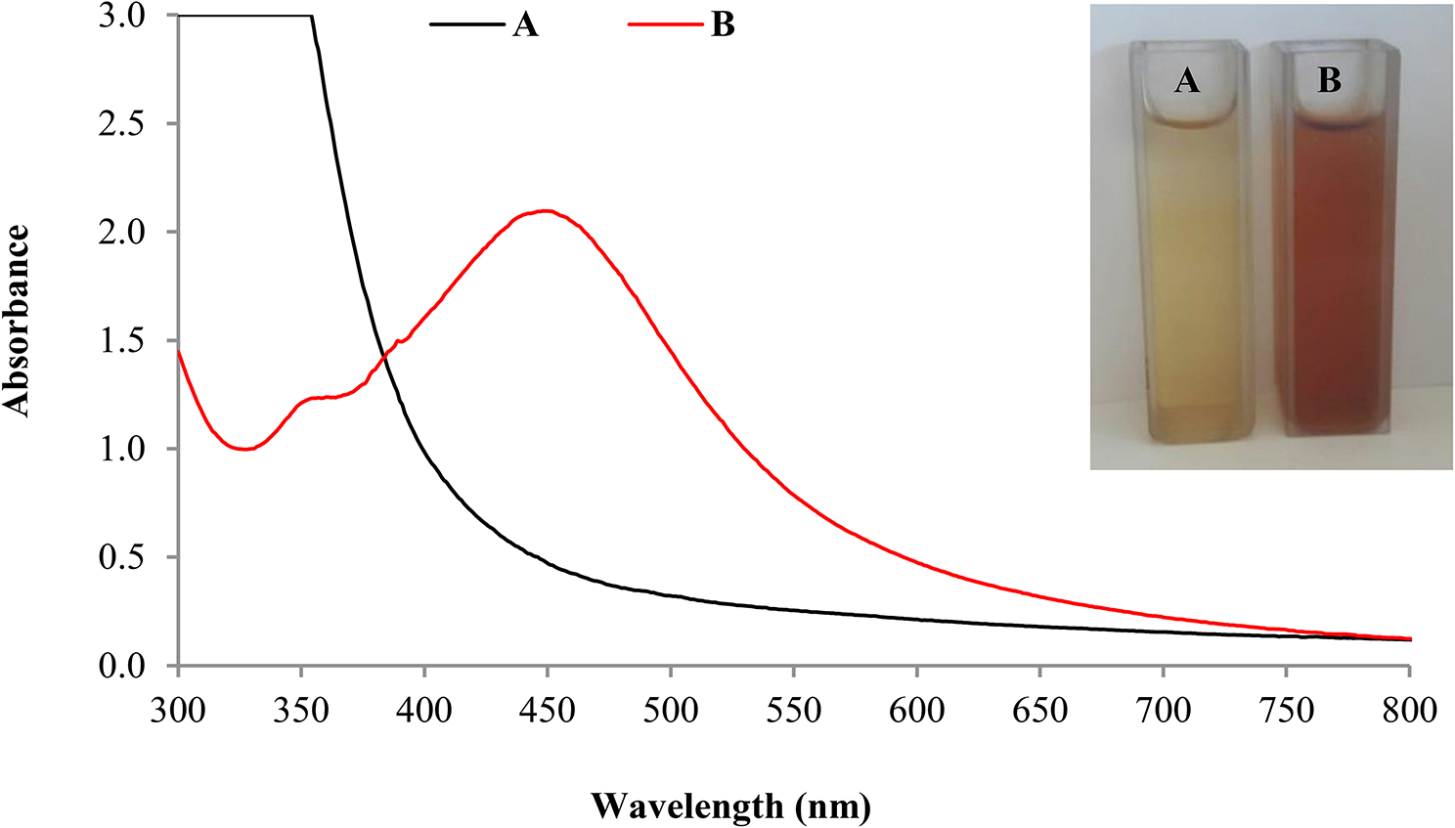
Color change and UV-visible spectroscopy of orange peel extract (**A**) and the resulted AgNPs (**B**)

Incubating orange peel aqueous extract prepared by boiling with AgNO_3_ for 24 h in dark resulted in color change of the reaction mixture from yellow to reddish brown (Fig. 5). This primarily indicated the formation of AgNPs. Spectral analysis of the diluted reaction mixture in the range of 300–800 nm revealed an absorption peak at 450 nm (Fig. 5). Similar results about color change and absorption peak of biogenic AgNPs were previously recorded in other studies [44]. Mediation of AgNPs synthesis by orange peel aqueous extract can be explained based on the reduction of silver cations of the precursor (AgNO_3_) into silver atoms by the peel extract that have potent reducing power. In addition, antioxidant activity of the peel extract helps in preventing re-oxidation of silver atoms into cations probably by the capping action. Similar explanation of AgNPs synthesis was formerly postulated [45]. Confirming AgNPs synthesis by absorption peak at 450 nm can be ascribed to surface plasmon resonance characteristic to AgNPs [46].

**Fig. 6:**
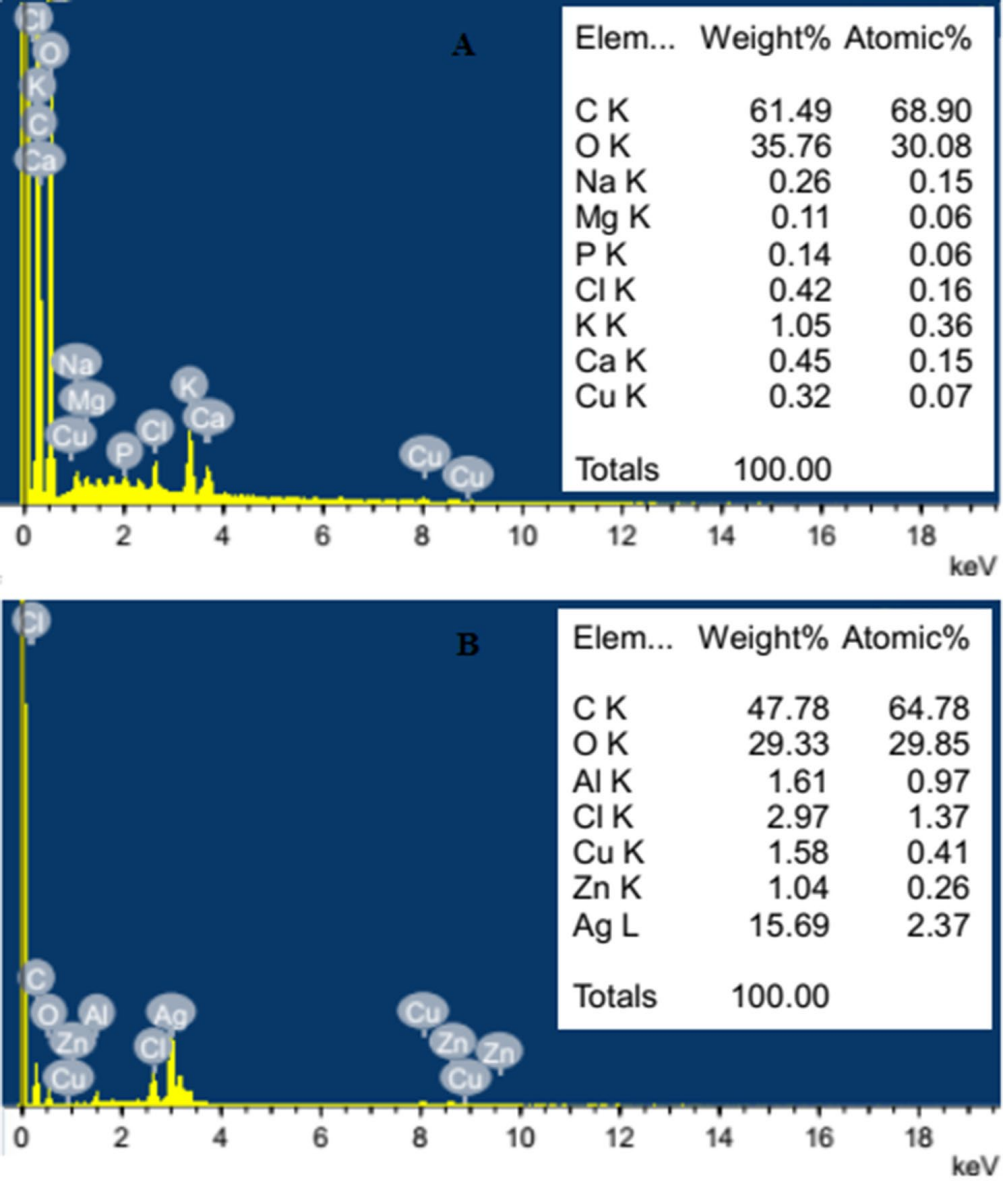
EDX analysis of orange peel extract (**A**) and the resulted AgNPs (**B**)

Energy-dispersive X-ray (EDX) analysis of orange peel extract prepared by boiling indicated that the bulk of elemental composition was carbon and oxygen (formed together about 97.25% weight composition). The remaining percent consisted of some alkali metals (such as sodium, potassium, calcium and magnesium) as well as traces of copper, phosphorus and chlorine (Fig. 6A). However, EDX analysis of the resulted AgNPs indicated dominance of silver (15.69% weight composition) (Fig. 6B).

**Fig. 7:**
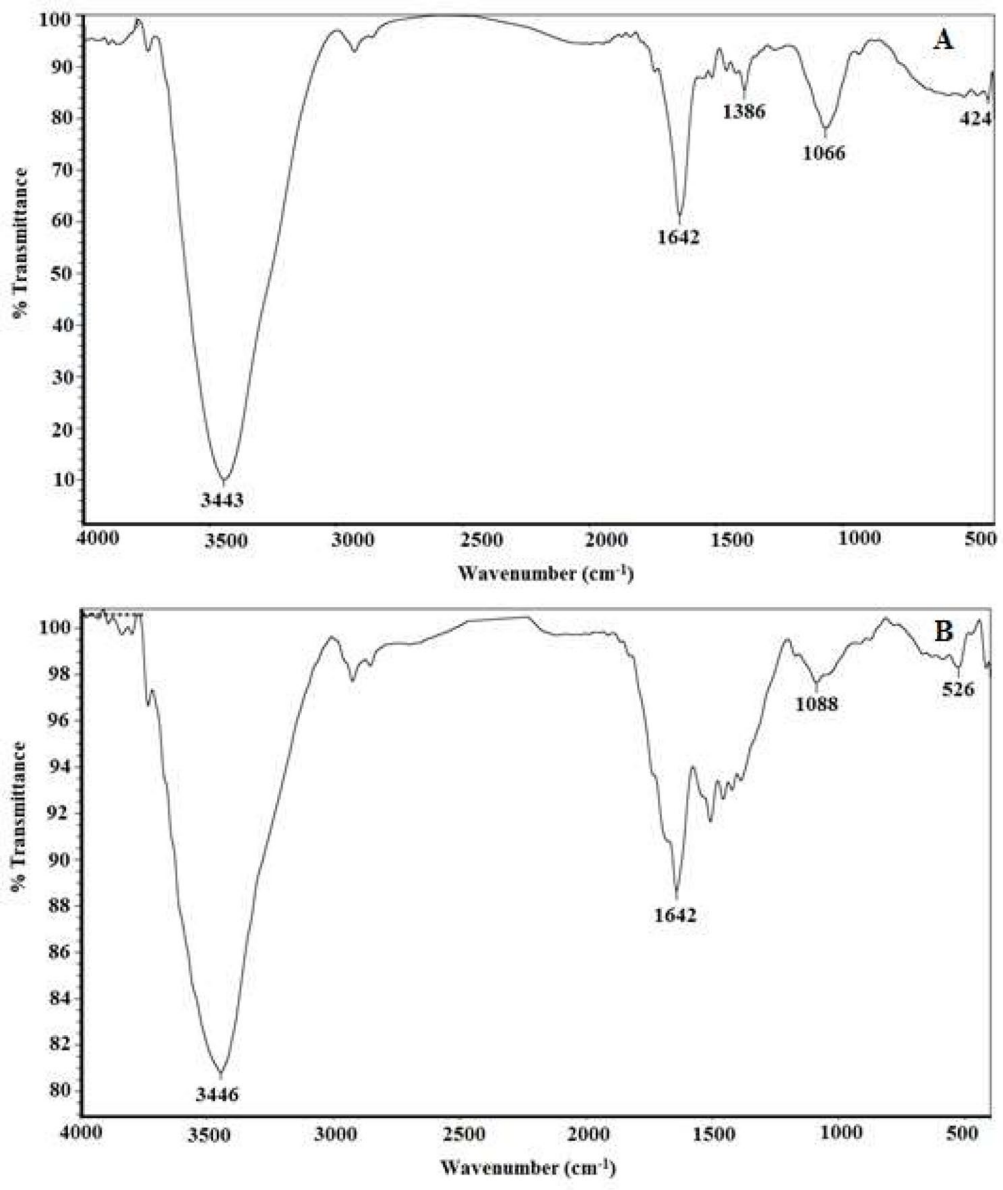
FTIR spectroscopy of orange peel extract (**A**) and the resulted AgNPs (**B**)

In addition, Fourier transform infrared (FTIR) spectroscopy of orange peel extract prepared by boiling revealed characteristic bands at 3443, 1642, 1386, 1066 and 424 cm^− 1^ (Fig. 7A). Sharp band at 3443 cm^− 1^ is corresponding to the stretching mode of inorganic hydroxyl groups (O-H) of water molecules [47]. This finding is logic since the extract prepared herein is aqueous. However, the band at 1642 cm^− 1^ is corresponding to the stretching vibration of olefinic double bond (C = C) of unsaturated fatty acids [48]. This finding is confirmed by GC-MS that revealed the presence of some oils (Table 1). Also, the band at 1386 cm^− 1^ is corresponding to the stretching mode of organic hydroxyl groups (C-O-H) of phenolic compounds [49]. This finding is confirmed by GC-MS that revealed the presence of three phenolic compounds with organic hydroxyl groups (Table 1). Moreover, the band at 1066 cm^− 1^ is corresponding to the stretching of carbonyl groups (C = O) of organic acids [50]. This finding is confirmed by GC-MS that revealed the presence of ascorbic acid (Table 1). In addition, the band at 424 cm^− 1^ is corresponding to the bending vibration mode of silicate groups (Si-O-Si) [51]. This finding is confirmed by GC-MS that revealed the presence of three silicone oils with silicate groups (Table 1). At the same times, characterization of the resulted AgNPs by FTIR revealed similar characteristic bands at 3446, 1642 and 1088 cm^− 1^ (Fig. 7B). This may indicate the contribution of unsaturated fatty acids and ascorbic acid present in orange peel extract as potent reducing and antioxidant agents in the synthesis and stabilization of AgNPs.

**Fig. 8:**
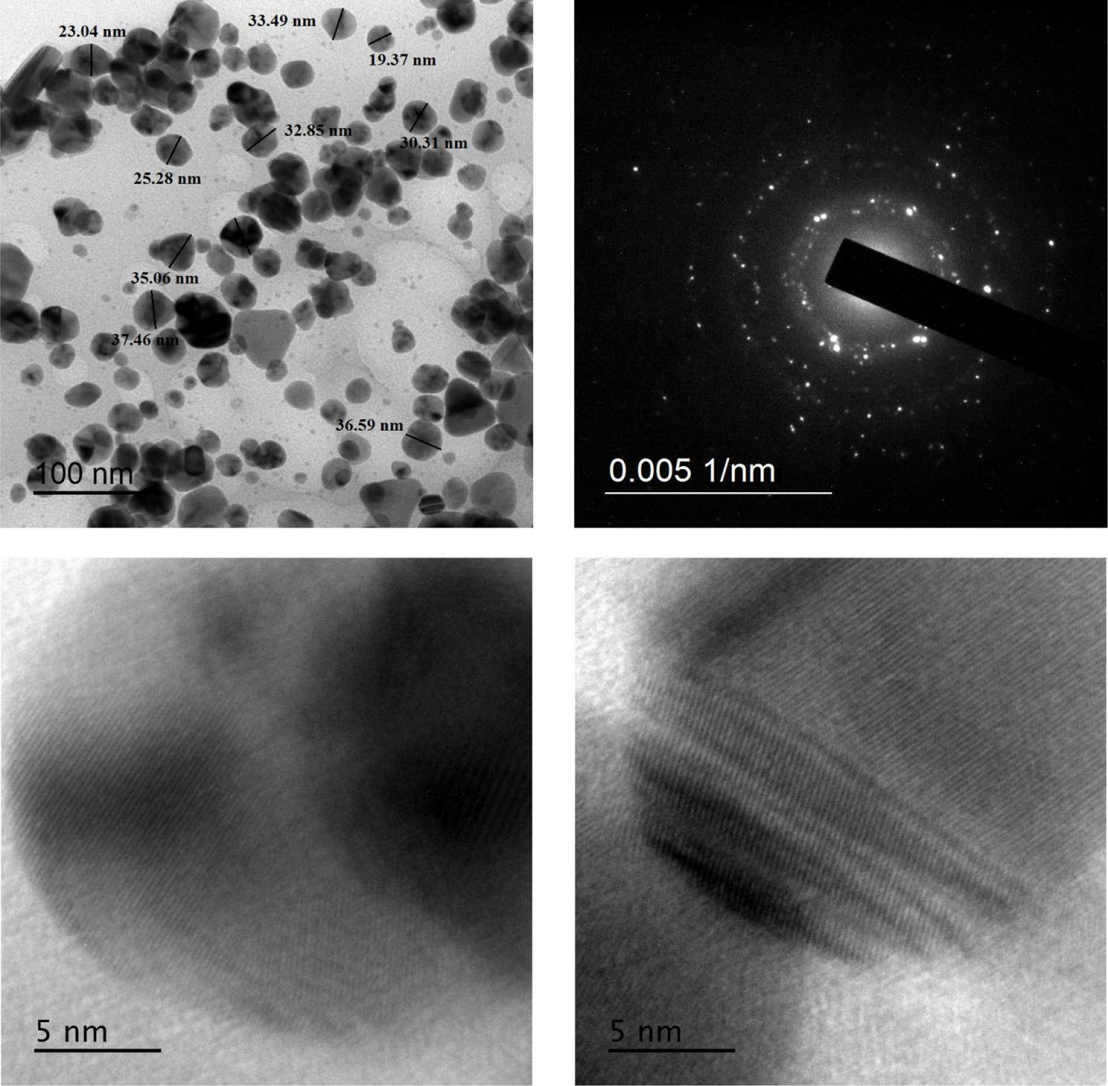
TEM and HR-TEM micrographs of AgNPs synthesized by the aid of orange peel extract

**Fig. 9:**
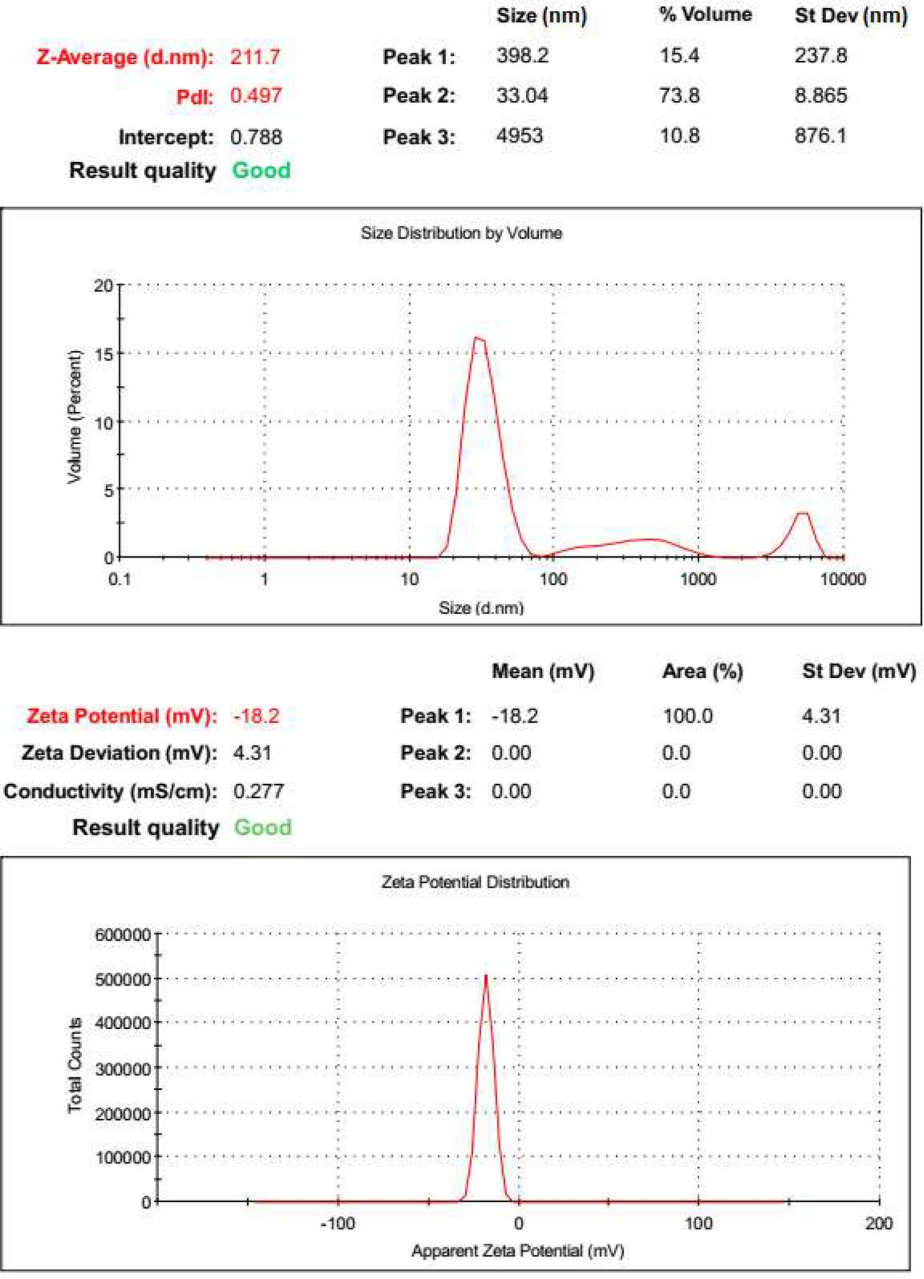
Zetametry of AgNPs synthesized by the aid of orange peel extract

Characterization of AgNPs by transmission electron microscopy (TEM) revealed almost spherical shape of the formed nanoparticles; and some were triangular (Fig. 8). TEM micrographs showed that AgNPs had size less than 100 nm (around 30 nm). High resolution TEM (HR-TEM) micrographs manifested lattice fringes of the formed AgNPs. In addition, the selected area electron diffraction of the formed AgNPs clearly indicated their crystalline nature (Fig. 8). Moreover, size distribution by volume of the biogenic AgNPs revealed that 73.8% of the formed nanoparticles had average size of 33.04 nm with low standard deviation value of 8.865 nm (Fig. 9). Out of the remaining volume, 15.4% of the formed nanoparticles had average size of 398.2 nm and only 10.8% had larger size. Also, size zetametry revealed a polydispersity index (PDI) of 0.497 referring to moderately uniform sample with respect to particle size. Furthermore, zeta potential distribution revealed that the formed AgNPs had zeta potential of -18.2 mV indicating high stability of these nanoparticles (Fig. 9). More or less similar properties of biogenic AgNPs were recently recorded [52].

### Yield, antioxidant potency and antitumor activity of AgNPs

Based on the determined concentration of AgNPs, about 82% of silver cations from AgNO_3_ were converted into silver atoms which self-assembled forming AgNPs (Table 2). This indicates the high efficiency of orange peel extract in mediating synthesis of AgNPs. Regarding their antioxidant capacity, the formed AgNPs could scavenge DPPH free radicals with IC_50_ of about 7 g 100 ml^− 1^ (Table 2). However, IC_50_ value recorded for AgNPs is higher than that recorded herein for orange peel aqueous extract prepared by boiling (about 2 g 100 ml^− 1^). This indicates that DPPH-scavenging ability of AgNPs is much lower than that of orange peel aqueous extract. Similarly, the formed AgNPs had potent reducing power and antioxidant activity (0.177 and 0.325 mg AAE ml^− 1^; respectively) (Table 2). However, these values are much lower than those recorded herein for orange peel aqueous extract (0.786 and 1.497 mg AAE ml^− 1^; respectively). Reasonable antioxidant capacity of AgNPs was recently proven via their potent free radical-scavenging ability, reducing power and antioxidant activity [53]. Antioxidant capacity of AgNPs can be attributed to elemental silver and/or the capping phytochemicals surrounding these nanoparticles [54].

**Table 2:** Yield, antioxidant capacity and antitumor activity of AgNPs synthesized by the aid of orange peel extract. Values listed are the mean of three replicates ± standard deviation

**- Yield**	
Concentration (ppm)	111 ± 9
Conversion (%)	82 ± 3
**- Antioxidant capacity**	
IC_50_ for DPPH-scavenging ability (g 100 ml^− 1^)	7.004 ± 0.098
Reducing power (mg AAE ml^− 1^)	0.177 ± 0.002
Antioxidant activity (mg AAE ml^− 1^)	0.325 ± 0.018
**- Antitumor activity**	
IC_50_ for HCT-116 (ppm)	16 ± 0.39
IC_50_ for HepG2 (ppm)	1.6 ± 0.02

**Fig. 10:**
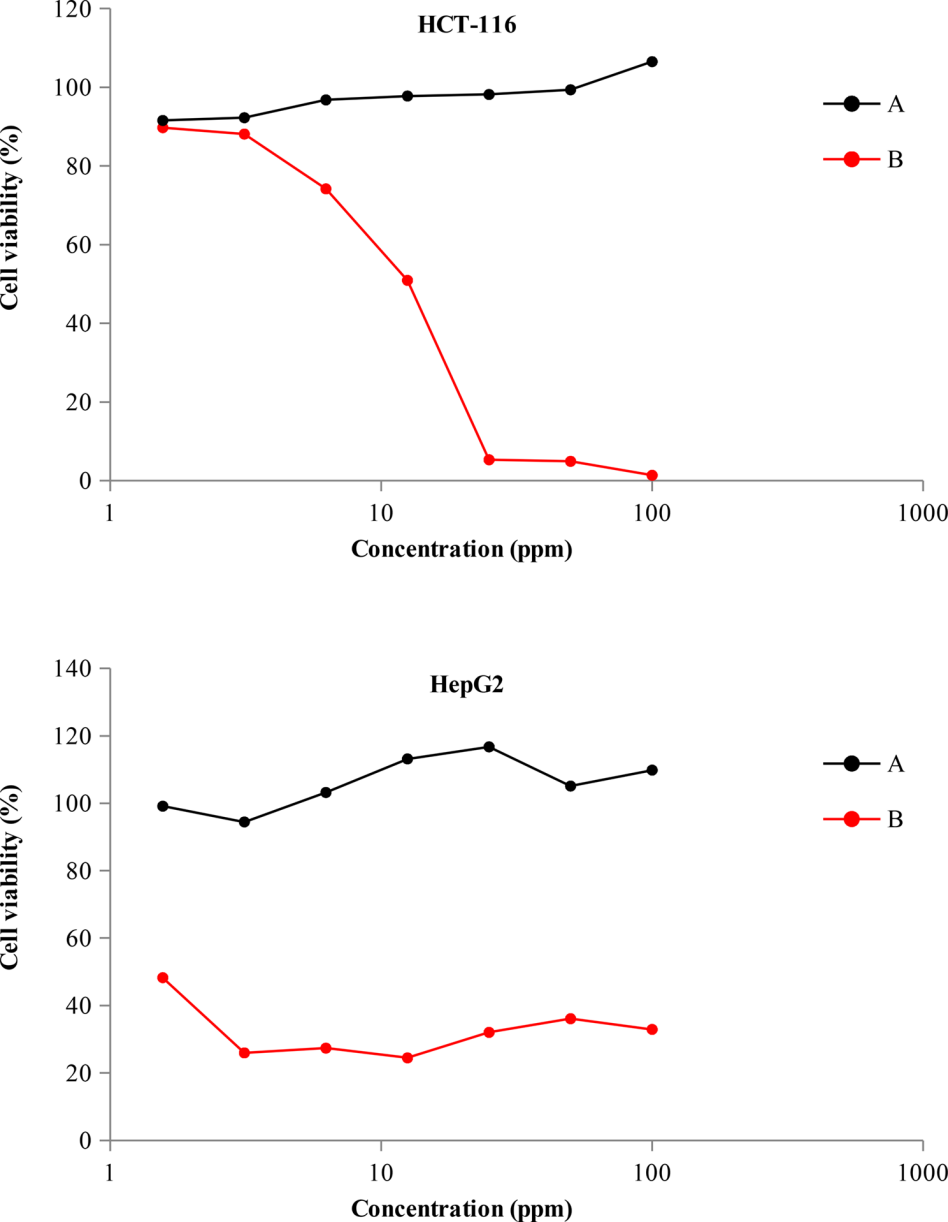
Antitumor activity of orange peel extract (**A**) and the resulted AgNPs (**B**) against two cell lines (HCT-116 and HepG2)

Though the peel extract prepared by boiling was non-toxic to the two cell lines (HCT-116 and HepG2), the formed AgNPs had marked cytotoxic effect on them (Fig. 10). Cytotoxic effect of AgNPs on HepG2 is much more pronounced than that on HCT-116. The recorded IC_50_ value of AgNPs for HepG2 is one-tenth that for HCT-116 (Table 2). Coinciding with these results, recent studies confirmed the strong antitumor activity of AgNPs. In this regard, biogenic AgNPs proved to have potent cytotoxic effect on different cell lines of breast cancer [55, 56]. The mechanisms proposed for antitumor activity of AgNPs include cell cycle arrest, induction of apoptosis and regulation of cytokine genes [57]. With respect to orange peel, recent studies documented antitumor activity of some bioactive compounds extracted from it [58]. Nevertheless, the safety of the aqueous extract recorded herein may be related to the solvent and its limited potency to extract antitumor compounds.

## Conclusion

The results obtained in the current study recommend using orange peel aqueous extract simply prepared by boiling for biogenic synthesis of AgNPs. The as-prepared AgNPs possess marked antioxidant and antitumor activities and can thus be considered for treating some oxidative stress- and tumor-related diseases. However, further studies on the effect of AgNPs on human normal cell lines are required.

## Data Availability

All data used in the current study are available within the manuscript.
